# Far-Infrared Based Pedestrian Detection for Driver-Assistance Systems Based on Candidate Filters, Gradient-Based Feature and Multi-Frame Approval Matching

**DOI:** 10.3390/s151229874

**Published:** 2015-12-21

**Authors:** Guohua Wang, Qiong Liu

**Affiliations:** School of Software Engineering, South China University of Technology, No. 382 Waihuan East Rd., Guangzhou 510006, China; w.guohuascut@gmail.com

**Keywords:** pedestrian detection, far-infrared video, advanced driver-assistance systems, gradient-based feature, candidate filters

## Abstract

Far-infrared pedestrian detection approaches for advanced driver-assistance systems based on high-dimensional features fail to simultaneously achieve robust and real-time detection. We propose a robust and real-time pedestrian detection system characterized by novel candidate filters, novel pedestrian features and multi-frame approval matching in a coarse-to-fine fashion. Firstly, we design two filters based on the pedestrians’ head and the road to select the candidates after applying a pedestrian segmentation algorithm to reduce false alarms. Secondly, we propose a novel feature encapsulating both the relationship of oriented gradient distribution and the code of oriented gradient to deal with the enormous variance in pedestrians’ size and appearance. Thirdly, we introduce a multi-frame approval matching approach utilizing the spatiotemporal continuity of pedestrians to increase the detection rate. Large-scale experiments indicate that the system works in real time and the accuracy has improved about 9% compared with approaches based on high-dimensional features only.

## 1. Introduction

Pedestrian detection is an important topic in different areas of computer vision, such as advanced driver-assistance systems (ADAS), video surveillance systems and autonomous robotics. Pedestrians are the most vulnerable traffic participants, because they are often seriously injured in traffic accidents. Nowadays, almost 1.2 million people are killed in traffic crashes every year, and 50 million people are injured around the world [1]. Therefore, it is necessary to explore robust and real-time pedestrian detection systems for the implementation of ADAS.

Far-infrared (FIR) pedestrian detection has already become a hot spot in recent research due to its potential application in both nighttime [2,3] and daytime [4,5] conditions. The major reasons are as follows: on the one hand, since a FIR camera can detect the amount of thermal radiation emitted from the scene [6] and does not depend on the illumination or color of the scene [7], it suits the detection of pedestrians better than the color camera and the near-infrared camera. On the other hand, the cost, size and weight of FIR cameras has kept decreasing these years [8,9], making them nowadays an interesting alternative to visible cameras for pedestrian detection systems [2,3,10]. As a result, FIR-based pedestrian detection has gained more and more interest in recent years [2,3,11].

Although many researchers have studied various approaches in recent years, a robust and real-time pedestrian detection system using a monocular vehicle-mounted FIR camera is still a challenging issue. This is because the detection approach must be able to deal with dynamic and complex street environments, the high variability of pedestrian size and appearance as well as the lack of texture information. Additionally, the complexity of the problem is increased by stringent accuracy criteria and superior real-time requirement in ADAS, so the development of a robust and real-time FIR-based pedestrian detection system remains a stark challenge.

Recently, many interesting approaches for FIR-based pedestrian detection have been proposed. On the one hand, most of them only utilize high-dimensional features to distinguish pedestrians from candidates, such as Pyramid Binary Pattern (PBP) [2], Pyramid Entropy Weighted Histograms of Gradients (PEWHOG) [3], Intensity Self Similarity (ISS) [12], and Histograms of Oriented Phase Energy (HOPE) [13] feature and so on. Filters based on low-dimension features to filter a large number of non-pedestrians efficiently have not been well developed. On the other hand, the current high-dimensional features for FIR-based pedestrian detection are not robust enough to deal with the enormous variance in pedestrians’ size and appearance. Accordingly, this paper proposes several approaches to deal with these two weaknesses.

The contributions of this paper are: (1) we designed two filters applying the low-dimensional features from pedestrians’ heads and the road, respectively, to efficiently reduce false alarms; (2) we propose a novel pedestrian detection feature encapsulating both the relationship of oriented gradient distribution and the oriented gradient code to deal with the enormous variance in pedestrians’ size and appearance; (3) we design a Multi-Frame Approval Matching (MFAM) approach that matches the filtered candidates with pedestrians of high confidence in order to increase the detection rate. As a consequence, we propose a robust and real-time pedestrian detection system based on a monocular FIR camera for ADAS. Experiments performed on large-scale FIR videos from various scenarios and seasons indicate that the proposed system is effective and promising. As the FIR camera perceives the amount of thermal radiation emitted from objects and does not depend on the illumination of the scene, the system can work during daytime and nighttime. Moreover, the system can detect pedestrians, bicyclists and motorcyclists within a range of 15–75 m away from the camera; while the detection range of the current state-of-the-art systems is limited by the visible light spectrum.

The remaining part of this paper is organized as follows: we review related work in Section 2. In Section 3 we provide a brief description about the proposed pedestrian detection system, and further details of each module are introduced in Section 4 and Section 5; more specifically, Section 4 describes how the candidates are generated by pedestrian segmentation and how the non-pedestrians are filtered by two proposed filters, and Section 5 describes the proposed classification based on a novel high-dimensional feature and a MFAM approach. We present our experiments in Section 6. In addition, some conclusions and implications for further research are described in Section 7.

## 2. Related Work

A considerable amount of previous work has addressed the problem of pedestrian detection. For a more comprehensive review of vision-based pedestrian detection, readers can refer to some recent survey papers [1,14,15]. Although most of the pedestrian detection approaches are based on color cameras, pedestrian detection using a monocular vehicle-mounted FIR camera has attracted increasing interest among the computer vision research community over the years.

Generally, the architecture of a FIR-based pedestrian detection system can be divided into three phases: candidate generation phase, feature extraction phase, and classification phase. An additional tracking phase could be also implemented for candidate refinement so as to augment the pedestrian detection rate as in [9,10,11]. The basic schedule of a FIR-based pedestrian detection system is to obtain image regions that are likely to contain pedestrians from every frame, and then feed the extracted features of every candidate to the pre-trained classifier to validate the pedestrians. We describe the related work of the three phases as follows.

The candidate generation phase tries to determine rectangle image regions that are likely to contain pedestrians, and this phase can be regarded as a rough classifier operated on the full-size image. Because the sliding window technique is usually not suitable for a system of low false alarm rate and is time consuming, the major candidate generation methods include intensity segmentation [16,17,18,19] and intensity-/variation-oriented projection [20,21]. Unfortunately, these approaches need to estimate an appropriate threshold, which is a key issue because the intensities of a pedestrian are usually non-uniform and the pedestrian may connect with some bright background objects. Other methods consist in detecting pedestrian heads based on pixel classification [22] and local sliding window technique based on key points [2,12]. Among these methods, intensity segmentation is more suitable than the other methods for generating candidates in FIR images, because pedestrians are usually warmer than the nearby environment, and hence they appear brighter than the adjacent background.

In the phase of feature extraction. The shape and appearance features are the most important cues for on-board pedestrian detection. The majority of FIR-based pedestrian detection systems use the Histogram of Oriented Gradients (HOG) feature [23] or a HOG-based feature. To name a few examples, Liu *et al.* [3] pointed out that the most informative components contained in HOG features are those extracted from the edge or contour regions of the pedestrian instead of the inner texture ones within the contour, and proposed a novel PEWHOG feature for FIR-based pedestrian detection. Instead of using the entropy to improve the well-known HOG feature [23], Gavrila *et al.* [24] noticed that FIR images are characterized by monotonic grey-level changes, and a new intensity-based feature called the Histogram of Local Intensity Differences (HLID) was introduced and is more suitable for the representation of FIR pedestrians than HOG. Besides, based on the traditional Local Binary Pattern (LBP) [25] feature, Sun *et al.* [2] encapsulated both the symmetry and spatial layout of texture cells, and proposed a novel PBP feature for FIR-based pedestrian detection. In order to observe the benefits augmented from the additional thermal channel, an extension of aggregated channel feature (ACF) [26] termed multispectral ACF feature is proposed in [27]. Generally, the abovementioned features have a quite excellent representation ability for FIR pedestrians in a small-scale pedestrian dataset but they are not robust enough for a practical pedestrian detection system, moreover, they are computation-intensive, especially when the number of candidates is large. Despite the various kinds of features that have been proposed, exploring more discriminative features for pedestrian representation has always been the pursuit, and the novel explored feature could potentially be complementary to the preceding ones.

Referring to the classification phase, traditional techniques like template matching [19] or those based on symmetry verification [28] are not precise enough for the task of pedestrian detection because of the different scales and high inter-class variation of pedestrians, so a mainstream approach is obtained through a machine learning algorithm based on the labeled training samples of pedestrians and non-pedestrians. Support Vector Machine (SVM) [12,13], AdaBoost [10,29], ensemble learning [11], and artificial neural networks [30], as well as different combinations or variations of them [10,31] have nowadays been adopted to find pedestrians in conjunction with the aforementioned features. Obviously, SVM is the most popular learning method and often produces accurate classifications with most of the features, and a linear kernel is efficient.

## 3. Overview of the Proposed Pedestrian Detection System

**Figure 1:**
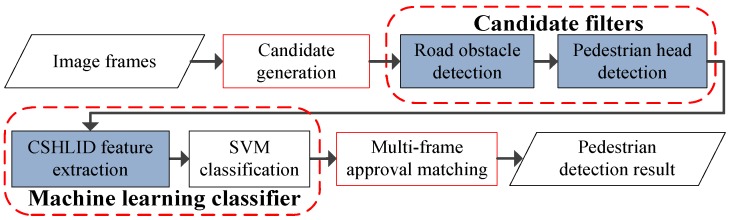
Overview of the system modules.

**Figure 2:**
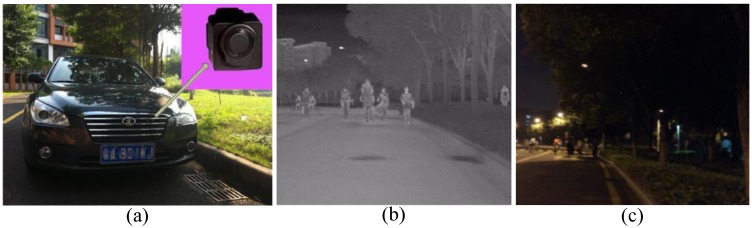
Data acquisition platform by monocular FIR camera and a comparison with a color image: (**a**) data acquisition platform by monocular FIR camera; (**b**) a FIR image; and (**c**) a color image.

The proposed system consists of four modules, which are denoted as candidate generation, candidate filters, machine learning classifier, and MFAM. The block diagram of the proposed pedestrian detection system is shown in Figure 1, where the four red rectangles denote the aforementioned four modules, and the three blue blocks denote the innovative contributions of our proposed system. The videos in our system are captured by a FIR camera fixed on the grille of an ordinary car, as shown in Figure 2a. An example of the captured image is shown in Figure 2b. Compared to the traditional visible spectrum, the far-infrared spectrum helps the pedestrians to stand out in the image when they are in dark environments—a comparison example is shown Figure 2b,c.

In the candidate generation phase, we utilize the image segmentation technique to generate the candidates because this generates much fewer candidates compared with the traditional sliding window approach. After extensive experiments, we found that the segmentation algorithm proposed in [10] has quite excellent segmentation accuracy, but it is time consuming when performed on the full-size image. Therefore, we perform this algorithm just on the odd rows of the full-size image, and the segmentation result of each even row is directly copied from the previous odd row’s. By this way, the computation load of the segmentation phase will be decreased by nearly 50%. Then, morphological operations are taken to refine the segmentation result.

The candidate filters reduce the number of the candidates for the subsequent classification module, and contain two proposed filters: pedestrian head detection filter and road obstacle detection filter. The pedestrian head detection filter detects a pedestrian’s head based on the fact that the head is the least likely body component to be occluded and is a relatively stable body component. During this procedure, we localize the head adaptively and propose a novel one-dimensional feature confusing brightness feature and gradient magnitude to represent the pedestrian head effectively. The main problem here is determining the parameter of the head classification threshold, as described in the following section. As for the road obstacle detection filter, it filters those candidates that don’t satisfy the ground plane constraint. In order to estimate the ground plane, we propose a method applying a quadratic curve to fit the bottom position of foreground segmentation. As a consequence, those non-pedestrians whose bottom positon is higher than the estimated ground plane can be filtered efficiently. Besides, these two filters are used in a cascaded fashion.

For the machine learning classifier, the main challenge is the required high performance and real-time constraints *versus* the large interclass variability in the pedestrian class. To deal with this problem we propose a three-branch classifier which is based on a novel feature to classify the pedestrians from among candidates. For the feature extraction, based on the newly proposed HLID feature in [24], the novel feature in this paper encapsulates both the relationship of oriented gradient distribution and the oriented gradient code. As for the learning algorithm, we use SVM to train the classifier with the training samples. As the size, pose and clothing of the pedestrians vary over a wide range, we train a three-branch SVM classifier based on the novel feature on three separated subsets containing samples of different size ranges. The three-branch SVM and novel feature help to reduce the classification complexity and improve the system accuracy.

In the MFAM phase. We utilize the prior knowledge that if a candidate has been classified as a pedestrian by the SVM classifier for several continuous frames, then the filtered candidate which is next to it in the next frame and of similar size will be directly regarded as a pedestrian too. With this in mind, multiple additional pedestrians can be re-detected so as to compensate for the errors in candidate classification. As a result, the pedestrian detection rate is further increased.

## 4. Candidate Generation and Candidate Filters

This section presents the details of the candidate generation and the candidate filter module. The candidate generation module is the first step of the proposed system. The candidates are obtained by a current segmentation algorithm proposed in [10] and morphological operations, and we do a little modification for the segmentation algorithm to speed up the system. Inspired by the importance of objectness measure mentioned in [32], in this section, we focus on the design of candidate filters so that a small set of candidates can be generated, and two filters are included, which are based on the pedestrians’ heads and the road, respectively.

### 4.1. Candidate Generation

**Figure 3:**
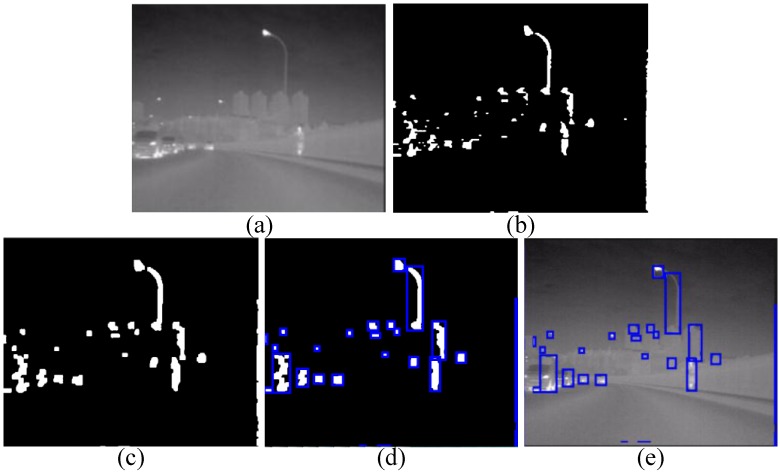
Example of candidate generation: (**a**) raw image; (**b**) result of interlaced segmentation; (**c**) result of morphological operations; (**d**) bounding boxes obtained from a connected component labeling algorithm; and (**e**) generated candidates.

This section introduces how we generate candidates. The traditional sliding window approach [33] will generate a large number of candidates, making it difficult satisfy the real-time requirement of ADAS. Alternatively, the segmentation-based technique can reduce the number of generated candidates significantly, thus making a substantial contribution to the real-time performance. That’s why we use the segmentation-based technique. Although FIR pedestrians are not always brighter than the background from a global perspective, a rather realistic assumption is that a FIR pedestrian appears brighter than the background from the view of a horizontal scan line [3]. Consequently, an adaptive dual-threshold segmentation algorithm is proposed to segment the image in [10]. However, this algorithm has to calculate two adaptive thresholds for every pixel, which is computationally expensive when performed on the full-size image. Therefore, we make a little improvement: we perform the segmentation algorithm just on the odd rows of the full-size image, and the segmentation result of each even row is directly copied from the previous odd row’s. Here we term the obtained segmentation algorithm as interlaced segmentation. In this way, the computation load of the algorithm will be reduced by nearly 50%. Details of the interlaced segmentation are done through the following two steps, and an example is shown in Figure 3a,b:

(1) For each pixel I(*i*, *j*) in the odd row of Figure 3a, we compute two thresholds, *i.e.*, a low threshold *T*_L_(*i*, *j*) and a high threshold *T*_H_(*i*, *j*) according to Equations (1) and (2) respectively, where *w* and α are set to 12 and 2 respectively, as in [10]:
  (1) $$T_{L}\left(i,j\right)=\sum_{x=i-w}^{x=i+w}I\left(x,j\right)/(2\times w+1)+\alpha$$
  (2) $$\begin{array}{l} T_{H}\left(i,j\right)=\max\left\{T_{1}\left(i,j\right),T_{L}\left(i,j\right)\right\},\\ T_{1}\left(i,j\right)=\min\left\{T_{2}\left(i,j\right),230\right\},\\ T_{2}\left(i,j\right)=\min\left\{T_{3}\left(i,j\right),T_{L}\left(i,j\right)+8\right\},\\ T_{3}\left(i,j\right)=\max\left\{1.06\times T_{L}\left(i,j\right)-\alpha ,T_{L}\left(i,j\right)+2\right\} \end{array}$$
(2) To segment the odd row of I(*i*, *j*) to be 1 or 0 according to Equation (3), where S(*i*, *j*) indicates the corresponding segmentation result. Then the segmentation result of each even row is directly copied from the previous odd row’s. The final segmentation results are shown in Figure 3b.
  (3) $$S\left(i,j\right)=\left\{ \begin{array}{l} 1,\;if\;I(i,j)>T_{H}(i,j)\\ 0,\;if\;I(i,j)<T_{L}(i,j)\\ 1,\;I(i,j)\in Others\;\&\;I(i-1,j)=1\\ 0,\;I(i,j)\in Others\;\&\;I(i-1,j)=0 \end{array}\right.$$

As one can see in Figure 3b, there are some noise segmentation results, and the segmentation results of the pedestrian are not connected. To deal with these two defects, the morphological operations: erosion and dilation with a mask of *M* × *N* pixels are applied to refine the segmentation results, and we will optimize *M* and *N* in the experiment par. An example of setting the size of the mask to be 3 × 3 is shown in Figure 3c. Not only have some noise segmentations been reduced, but the pedestrian is also connected correctly, so this step is beneficial to refine segmentation results in cluttered scenes. Then, all the bounding boxes of the connected regions in Figure 3d are used to generate candidates from Figure 3a, the generated candidates are shown as the blue rectangle regions in Figure 3e.

### 4.2. Pedestrian Head Detection Filter

In this section, we propose a novel pedestrian head detection approach to pre-classify (*i.e*., filter) the candidates. The reasons we focus on pedestrians’ heads are that we note that the FIR pedestrian head is the least likely body component to be occluded and it is a relatively stable body component. The proposed head detection approach is composed of three steps, which are denoted as adaptive head location, head feature extraction, and head classification:

(1) The adaptive head location module locates the pedestrian head adaptively on a candidate based on the analyzing of a pixel-intensity vertical projection curve. As a result, only the bright image region in the upper position of a candidate will be considered whether it contains a pedestrian head or not;
(2) In the head feature extraction step, we propose to fuse both the brightness image and gradient magnitude image to enhance the representation of the pedestrian head. For the sake of efficiency, only a one-dimensional feature is extracted;
(3) In the head classification step, since the dimension of the head feature is one, the main problem is determining the parameter of the head classification threshold, as described in the following section.

**Figure 4:**
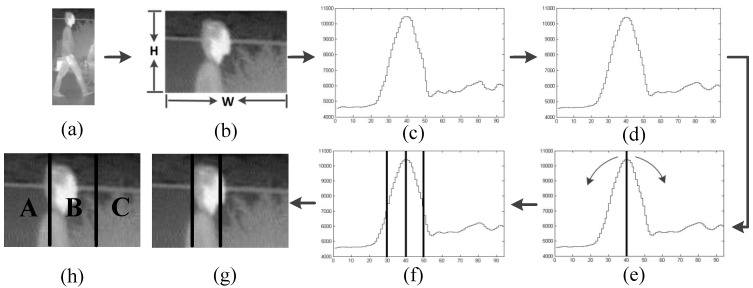
Schematic diagram of the adaptive head location module and a comparison with the fixed location of the pedestrian head: (**a**) a candidate; (**b**) the cropped 1/5 image region of a candidate; (**c**) the pixel-intensity vertical projection curve; (**d**) the smoothed curve; (**e**) search from the maximum value of the projection curve; (**f**) the position of the stripe in the projection curve; (**g**) the position of the stripe in the crop image; and (**h**) an example of fixed location of the pedestrian head.

As shown in Figure 4a–g, the adaptive head location module for each candidate is done through the following three steps:

(1) Pixel-intensity vertical projection. For a candidate, as shown in Figure 4a, we crop the top 1/5 of the image region, as shown in Figure 4b, and compute the vertical projection curve *V*(*x*) according to Equation (4). The result is shown in Figure 4c, where the maximum value usually corresponds to the center position of the pedestrian head:
  (4) $$V(x)=\sum_{y=0}^{N-1}f(x,y)$$
  where *f*(*x*, *y*) denotes the intensity value of a pixel at (*x*, *y*) of Figure 4b, and *V*(*x*) is the vertical projection curve.
(2) Noise reduction. Reduce the noise of *V*(*x*) according to Equation (5) and get a smoothed one, *V*_s_(*x*). The smooth window size *n* is set to 5 experimentally. The result is shown in Figure 4d:
  (5) $$V_{s}(x)=\sum_{k=a}^{b}V(k)/n,\;a=x-\left\lfloor n/2\right\rfloor ,\;b=x+\left\lceil n/2\right\rceil -1$$
  where *n* denotes the size of the smoothing window.
(3) Stripe location. *i.e.*, to locate a stripe that probably contains pedestrian head. Starting from the maximum value of *V*_s_(*x*), we search the maximum raising point on the left (*P*_l_) and the maximum falling point on the right (*P*_r_) according to Equations (7) and (8), respectively, as shown in Figure 4e. Then [*P*_l_, *P*_r_] is the obtained strip, as shown in Figure 4f, the obtained position of the stripe in the cropped image is shown in Figure 4g:
  (6) $$V_{s}^{\prime }(x)=dV_{s}(x)/dx=V_{s}(x+1)-V_{s}(x)$$
  (7) $$P_{l}=\underset{x}{\arg\max}V_{s}^{\prime }(x),\;x\in [x_{l},x_{c}]$$
  (8) $$P_{r}=\underset{x}{\arg\max}V_{s}^{\prime }(x),\;x\in [x_{c},x_{r}]$$
  where *x*_c_ denotes the abscissa position of the maximum value of *V*_s_(*x*), *P*_l_ and *P*_r_ denote the search result on the left and on the right respectively, *x*_l_ and *x*_r_ denote the search range to the left and to the right direction respectively, and [*x*_l_, *x*_c_] = [*x*_c_, *x*_r_] = *w*/2, where *w* is the width of the candidate.

As shown in Figure 4g, our adaptive head location method is more accurate at locating the head region of the pedestrian than the method proposed in [34] that simply uses a Haar-like template to locate the head fixedly, as shown in Figure 4h.

**Figure 5:**
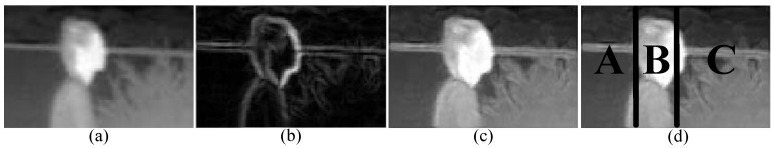
Enhanced head information: (**a**) brightness image; (**b**) gradient image; (**c**) combination of brightness image and gradient image; and (d) the obtained strip in Figure 4g.

In the head feature extraction step, the method proposed in [34] only uses the brightness feature to represent the pedestrian’s head, which is not robust enough. Inspired by [32], we propose to fuse the brightness feature and gradient magnitude feature by Equation (9) to enhance the representation of the pedestrian’s head, so that an enhanced image can be obtained. An example is shown in Figure 5, where the pedestrian is enhanced because the gradient magnitudes on pedestrian region is abundant. Then, based on the enhanced image in Figure 5c and the obtained strip in Figure 4g, we extract the head feature *H*_f_ according to Equation (10):

(9) $$\begin{array}{l} I_{f}(i,j)=\min[I(i,j)+I_{g}(i,j),255],\\ I_{g}(i,j)=abs[I(i,j-1)-I(i,j+1)]+abs[I(i-1,j)-I(i+1,j)] \end{array}$$

where *I* denotes the raw image, *I*_g_ denotes the gradient magnitude image, and abs (•) denotes the absolute value:

(10) $$H_{f}=mean(A+C)-mean(B)$$

where *A*, *B* and *C* denote the sum of brightness in the corresponding image region of Figure 5(d), respectively, and mean (•) denotes the mean value.

As the head feature *H_f_* is a one-dimensional feature, in order to perform head classification, the classification threshold is the only parameter that needs to be considered. *i.e.*, once the *H_f_* is higher than the classification threshold, the corresponding candidate will pass the pedestrian head detection filter. We will optimize the classification threshold in the experiment section (Section 6.3). Besides, the contribution of the adaptive head location and the contribution of additional gradient magnitude image will also be evaluated in the experiment section (Section 6.3).

### 4.3. Road Obstacle Detection Filter

**Figure 6:**
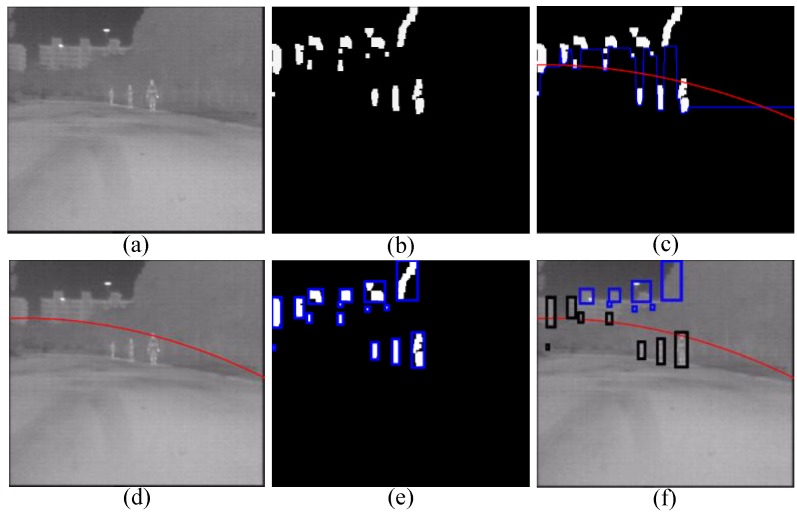
Schematic diagram of road obstacle detection filter: (**a**) raw image; (**b**) result of interlaced segmentation; (**c**) the bottom position of the foreground segmentation (the **blue** line) and the fitted quadratic curve (the **red** curve, *i.e.*, the estimated ground pane); (**d**) the estimated ground pane on the raw image; (**e**) bounding boxes of generated candidates; and (**f**) filtered non-pedestrians (**blue** rectangles) and detected road obstacles (**black** rectangles).

After pedestrian head detection filter is applied, in this section we propose a novel road obstacle detection filter to further filter the candidates by using the scene context information from the road. More specifically, through the estimation of the road obstacle bottom position (corresponding to the ground plane), this filter can filter those candidates whose bottom positions are higher than the ground plane, because those pedestrians on the ground plane are the objects we should concern in ADAS. The procedure of this filter is shown in Figure 6. Firstly, we search from the bottom of the segmented image in Figure 6b, and record the vertical coordinates of the first foreground pixel on each column, and record the obtained coordinates in an array named *G*. By using a high threshold *T* to truncate *G*, we obtain the Bottom Position of the Foreground Segmentation (BPFS), as the blue line shown in Figure 6c. More specifically, the truncation is done as follows: if *G_i_* is higher than *T*, *G_i_* is set to be *G_i_*_−1_, and *T* is set to be 4/5 of the height of the full-size image, which is much higher than the position of actual road plane. Secondly, we propose applying quadratic curve to fit the BPFS, so that more non-pedestrians can be filtered. As shown in Figure 6c, where the fitting curve (red curve) is lower than the BPFS (blue line) on the whole, as a consequence, those non-pedestrians whose bottom positons are higher than the estimated red curve can be filtered efficiently, like the filtered blue rectangles in Figure 6f. Then, the remaining black rectangles in Figure 6f are the detected road obstacles, which will then be fed to the machine learning classifier.

**Figure 7:**
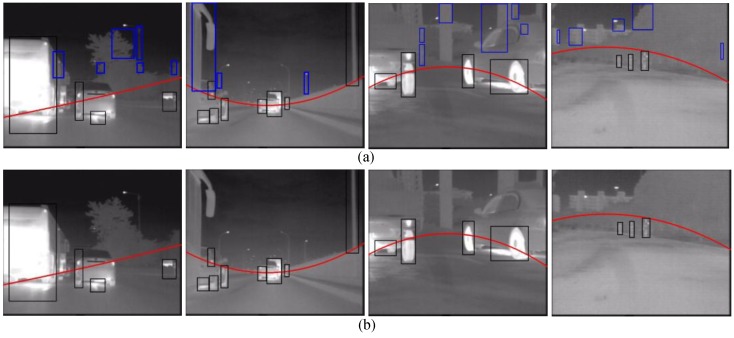
Examples of road obstacle detection filter: (**a**) all the candidates (rectangle image regoions) before filtering by the road obstacle detection filter, the **red** line is the estimated ground pane and the **blue** rectangles are the filtered candidates; and (**b**) all the candidates (**black** rectangle image regions) after filtering by the road obstacle detection filter.

Some examples of road obstacle detection filter are shown in Figure 7, which show that the typical non-pedestrians (such as tree crowns, utility poles, parts of buildings and so on) can be filtered by this filter.

## 5. CSHLID-Based SVM Classification and Multi-Frame Approval Matching

Key components of a pedestrian detector are the pedestrian features and the machine learning algorithm employed to obtain the detector [35]. This section introduces the details of our proposed coded similarity HLID (CSHLID) feature, the design of the three-branch SVM classifier, and the MFAM module. The CSHLID feature is an improved version of the newly HLID feature [24] which is specifically designed for FIR pedestrians. The SVM is used to train the CSHLID feature, and three branches are included to reduce the complexity of the classifier and to improve classification accuracy, so that a CSHLID-based three-branch SVM classifier is obtained. After this classifier has classified the filtered candidates, we introduce a MFAM approach to further increase the detection rate.

### 5.1. CSHLID-Based Classification

**Figure 8:**
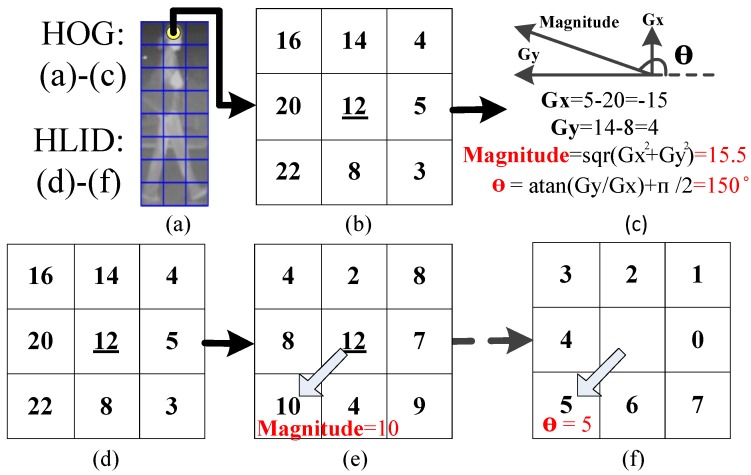
Conceptual differences of HOG and HLID feature with eight neighbors. (**a**) an example of cell partition within a candidate; (**b**) pixel values of a 3 × 3 image region; (**c**) the calculation of gradient magnitude and gradient angle when calculating HOG feature; (**d**) pixel values of a 3 × 3 image region; (**e**) the calculation of gradient magnitude when calculating HLID feature; and (**f**) the calculation of gradient angle when calculating HLID feature.

To extract a robust feature that discriminates pedestrians from the non-pedestrians is a paramount step, because the performance of a classifier mainly depends on the adopted features. Numerous features have been proposed for pedestrian detection in recent years. The HLID feature, recently proposed by [24], has properties that favor its usage in FIR pedestrian representation such as discriminative power, computational simplicity, and tolerance against monotonic gray-scale changes of FIR pedestrians. More specifically, HLID is an improved version of the well-known HOG feature [23] for FIR pedestrian representation. Based on HOG, for the estimation of gradient magnitude, HLID uses the maximum absolute intensity difference between the center pixel and the neighboring pixels instead of the magnitude (as shown in Figure 8e), and for the gradient orientation, HLID uses the neighboring pixel position instead of θ obtained from *Gx* and *Gy* (as shown in Figure 8f). Figure 8 shows an illustration with eight neighbors of HOG and HLID respectively, where the main conceptual differences are the manner of computing the magnitude and the angle θ of every pixel.

**Figure 9:**
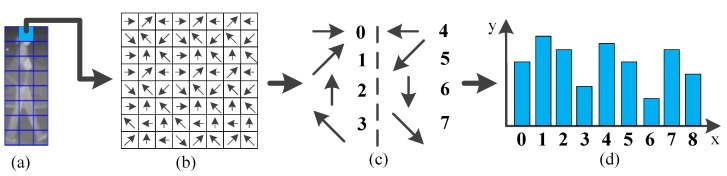
Schematic diagram of the histogram of maximum gradient oriented for one cell in HLID feature extraction manner. (**a**) an example of cell partition within a candidate; (**b**) the distribution of gradient orientation within a cell; (**c**) the coding of gradient orientation; and (**d**) the histogram of gradient orientation.

**Figure 10:**
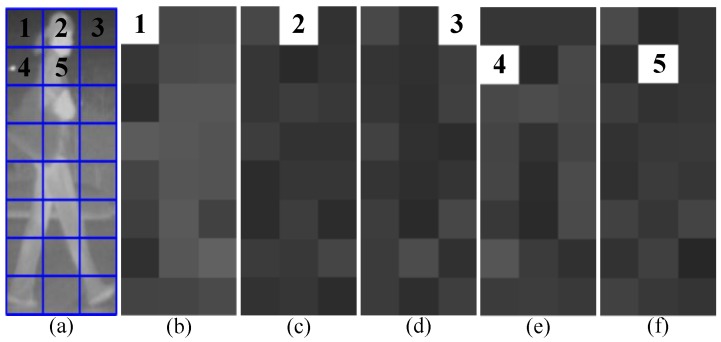
Visualization of cell similarity using histogram difference, computed at position 1–5 for some examples. A brighter cell shows a higher degree of similarity. (**a**) an example of cell partition within a candidate, and the first five cells are denoted by number 1–5; (**b**) cell similarity between the first cell and the other cells; (**c**) cell similarity between the second cell and the other cells; (**d**) cell similarity between the third cell and the other cells; (**e**) cell similarity between the fourth cell and the other cells; and (**f**) cell similarity between the fifth cell and the other cells.

Based on HLID, a new gradient-based feature named CSHLID is proposed in this paper. The CSHLID feature is a modified version of the HLID [24] feature. The underlying idea of the novel feature is that the HLID feature hasn’t considered the relationship of oriented gradient distribution or the code of oriented gradient, so we encapsulate these two kinds of information for the HLID feature, and propose the CSHLID feature. *i.e.*, directly applying HLID feature does not capture the spatial relationship of the texture cells (an image region of 8 × 8 pixels) and spatial layout of gradient orientation of the candidate which are important for FIR pedestrian representation. Armed with this idea, we improve the HLID feature as follows:

On the one hand, to capture the spatial relationship of texture cells within a candidate, we compute the cell similarity of every two cells features obtained from HLID feature extraction. The feature extraction scheme of one cell by the HLID feature extraction method is shown in Figure 9 and the similarity *h* is measured by Equation (11). The cell similarity is visualized in Figure 10. 

**Figure 11:**
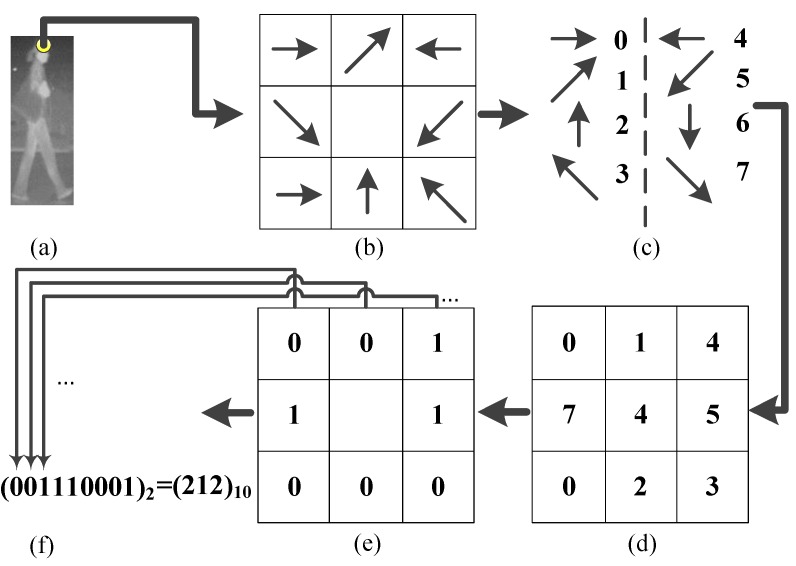
Schematic diagram of gradient direction of LBP coding on a pixel. (**a**) an example of a candidate; (**b**) the distribution of gradient orientation around the pixel; (**c**) the coding of gradient orientation; (**d**) the coding result of the pixel and its surrounding pixels; (**e**) the comparision results of the pixel and its surrounding pixels; and (**f**) the binary-to-decimal conversion.

We find that the oriented gradient distribution relationship can represent the relationship of different part patterns of a pedestrian. After all the similarities have been computed and normalized by Equation (12), we name the obtained feature as the Gradient-Similarity feature. On the other hand, to capture the spatial layout of gradient orientation of the candidate, we code the gradient orientation of every pixel in a LBP operator manner. As shown in Figure 11, where Figure 11c is the corresponding position pattern of gradient orientation, Figure 11d–f are the scheme of the LBP operator on a pixel, and Figure 11f is the result of LBP coding for a pixel, which will give a histogram feature. Then the histogram is normalized by Equation (13). We name the obtained feature the Gradient-LBP feature. As a result, our CSHLID is the concatenation of HLID, Gradient-Similarity and Gradient-LBP feature. The additional Gradient-Similarity and Gradient-LBP feature are computed based on the HLID feature and their computation load is low, so we can obtain richer representations without paying a heavy computation price. The improvement of the CSHLID feature and a comparison with the state-of-the-art pedestrian detection features will be tested on the experiment part.

(11) $$h=\sum_{k}^{k=8}\left|h_{1}[k]-h_{2}[k]\right|$$

where *h*_1_[*k*] and *h*_2_[*k*] are the histogram features of two cells within a candidate.

(12) $$h_{s}[i]=\sqrt{h[i]/\sqrt{\sum_{i=1}^{d_{1}}{\left(h[i]\right)}^{2}+10^{-5}}}$$

where *h*_s_[*i*] is the normalized Gradient-Similarity feature, and *d*_1_ is its feature dimension.

(13) $$h_{l}[i]=\sqrt{H_{i}/\sum_{i=1}^{d_{2}}{\left(H_{i}\right)}^{2}+10^{-5}}$$

where *h_l_*[*i*] is the normalized Gradient-LBP feature, and *d*_2_ is its feature dimension.

As for the machine learning algorithm, currently, SVM and variants of boosted decision trees are the two leading machine learning algorithms for FIR-based pedestrian detection due to their superior performance and efficiency. SVM is able to learn in sparse, high-dimensional space with relatively few training samples, so it is chosen as the machine learning algorithm in our system. For the sake of real-time constraints, the linear kernel is adopted. We utilize a Libsvm tool [36] to implement the training of the detector, and the penalty factor *C* is optimized by 10-flod validation on the corresponding training set, and the final value is different from 1000 as being used in [29]. For the sake of reducing the inner variance of the pedestrians and non-pedestrians, all the candidates are divided into three kinds of distances ((20,48], [48,72) and [72,+] pixel) according to their heights, and they are all resized to 64 × 24 pixels by using a bilinear interpolation algorithm. Since the candidates are divided into three kinds of distances, three-branch CSHLID-based lin-SVM are trained, respectively.

### 5.2. Multi-Frame Approval Matching

**Figure 12:**
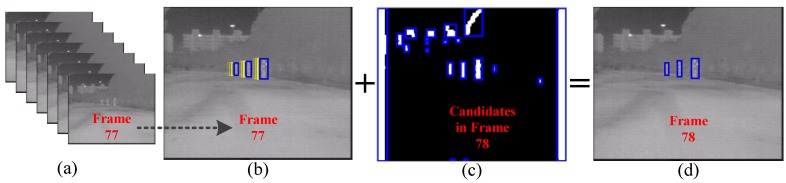
Schematic diagram of multi-frame approval matching: (**a**) video sequence; (**b**) three pedestrians that satisfied the multi-frame approval; (**c**) candidates in the next frame; and (**d**) additional detections obtained by matching the blue rectangles in (**b**,**c**).

The two candidate filters can decrease the false alarm rate, but they don’t make any contribution to increasing the pedestrian detection rate. This section utilizes the prior knowledge that if a candidate has been classified as pedestrian by the SVM classifier for several continuous frames, then the filtered candidate next to it in the next frame which is of similar size will be directly regarded as a pedestrian too, and we name this process the MFAM algorithm. A schematic diagram of MFAM is shown in Figure 12. It makes full use of those pedestrians who are segmented successfully but are misclassified by the classifiers, which is simple and of much lower computational load compared with traditional tracking approaches (Kalman filtering [10] or particle filter [11]), and can help to increase the detection rate of the system. However, it may increase some false alarms if a non-pedestrian is classified as pedestrian incorrectly for several continuous frames. Fortunately, this case happens only very rarely after the candidate filters have been designed to suppress the false alarms.

More specifically, the MFAM algorithm consists of two steps. Firstly, those candidates which have positive outputs by means of CSHLID-based SVM classifier are selected, then the continual detection time and miss detection time of them are calculated respectively. If the continual detection time is more than 3, then the candidate will be considered as a pedestrian of high confidence; once the miss detection time is more than 15, then the pedestrian will be removed from the pedestrian list. Secondly, each pedestrian of high confidence will be matched with the filtered candidates in the next frame following the nearest neighbor matching rule of Equation (14). If a candidate in the next frame is matched, then it will be regarded as a pedestrian directly:

(14) $$\left|x_{1}\text{-}x_{2}\right|<T\&\left|y_{1}\text{-}y_{2}\right|<T\&\left|w_{1}\text{-}w_{2}\right|<T\&\left|h_{1}\text{-}h_{2}\right|<T$$

where *w*_1_ and *h*_1_ represent the width and height of the bounding box of the first candidate respectively, whose center coordinate is (*x*_1_, *y*_1_); in the same way, *w*_2_, *h*_2_ and (*x*_2_, *y*_2_) are the second candidate’s. *T* is the matching threshold which will be optimized in the following experiments part (Section 6.5).

## 6. Experiments

In this section, we conduct experiments to answer the following questions: (1) how is the performance of the pedestrian head detection filter and which is the optimal classification threshold for this filter? (2) How’s the accuracy performance of the state-of-the-art features and our proposed CSHLID feature for FIR pedestrian classification? (3) What are the optimal parameters for the morphological operations, SVM and MFAM? (4) How is the accuracy performance of the proposed pedestrian detection system and the related systems? (5) How’s the real-time performance of related pedestrian detection systems?

### 6.1. Dataset

**Figure 13:**
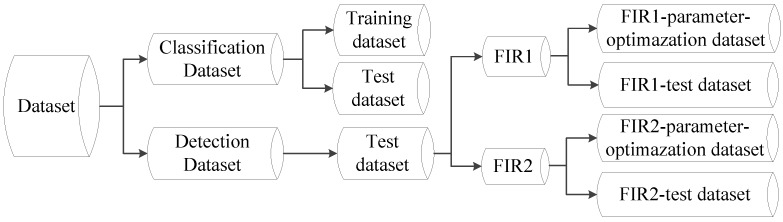
Framework of the datasets used in the experiments. The datasets consist of two parts: Classification Dataset and Detection Dataset. Classification Dataset: this dataset consists of a set of cropped images of pedestrians and non-pedestrians, which contains training set and test set, is used for training a classifier and classification performance evaluation respectively. Detection Dataset: this dataset consists of full-size images and annotations indicating the position and size of the pedestrians, which contains test set only, is used for parameter optimization and detection performance evaluation, and FIR1 refers to self-acquired videos, FIR2 refers to all the test videos from the detection part of LSIFIR [37] benchmark dataset.

Our experimental datasets included two sources: a benchmark LSIFIR dataset [37] and a dataset built by us (a self-acquired dataset). The LSIFIR dataset is captured by a monocular vehicle-mounted FIR camera with a resolution of 164 × 129 pixels in urban environments, and the self-acquired dataset was captured by a monocular vehicle-mounted FIR camera with a resolution of 352 × 288 pixels in urban and rural environments. The composition framework of our datasets used in the experiments is shown in Figure 13.

**Figure 14:**
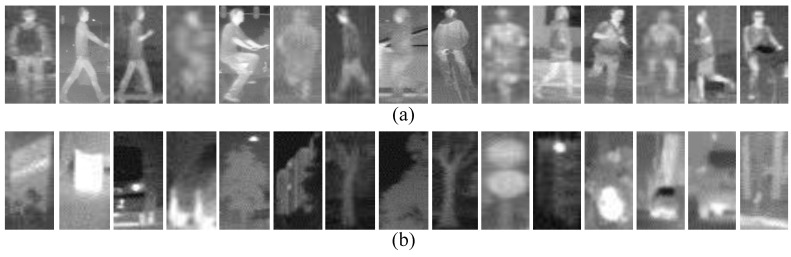
Examples from the Classification Dataset: (**a**) examples of pedestrians; and (**b**) examples of non-pedestrians. For visualization purpose, the examples have been resized to the uniform spatial resolution.

**Figure 15:**
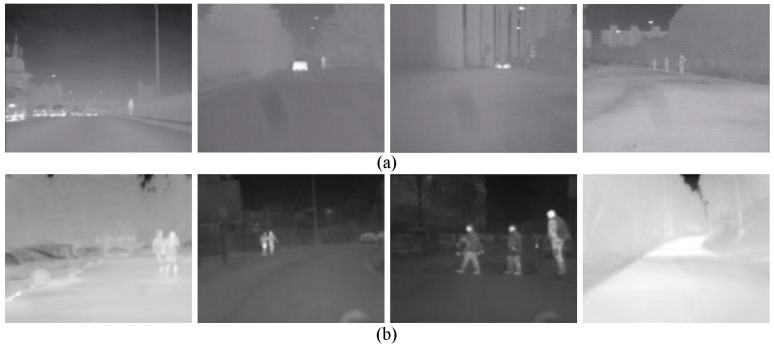
Snapshots of the Detection Dataset: (**a**) examples from FIR1 dataset; and (**b**) examples from FIR2 dataset.

The details of each component of the datasets are as follows:

- Classification Dataset. This dataset consists of training set and test set. The training set consists of 23,603 one-channel samples cropped from the self-acquired videos, 5963 of which are positive samples and 17,640 are negative samples. In order to decrease the inner variance of the samples, all of them are further divided into three disjoint subsets corresponding to three kinds of distances to the camera according to the heights of the samples, and each subset is scaled to a uniform spatial resolution. The number of positive samples in near, middle and far distance are 1084, 1701 and 3178, respectively, and the negative samples in near, middle and far distance is 4790, 3098 and 9752, respectively. The test set is used to evaluate the performance of a pedestrian classifier on pedestrian classification (classifier-level), and consists of 4800 pedestrian and 5000 non-pedestrian FIR samples. Most of the pedestrian samples are pedestrians in up-right posture, but the bicyclists and motorcyclists are also included as they are vulnerable participants of the road users. Some examples from the Classification Dataset are shown in Figure 14.

- Detection Dataset. The Detection Dataset is used to evaluate the performance of a pedestrian detection system on pedestrian detection (system-level) and optimize parameters. This dataset only contains test set and consists of two parts: self-acquired videos (FIR1) and all the test videos from the detection part of LSIFIR [37] (FIR2). (1) The FIR1 is captured in 11 different road sessions, corresponding to 11 video sequences in total and each of which contains different numbers of frames. It contains 3940 one-channel full-size images and 1511 pedestrians in total. Amongst the 11 video sequences which are named as FIR1-seq01 to FIR1-seq11 in Table 1, 3 video sequences (FIR1-parameter-optimazation dataset) are randomly selected for optimizing parameters, and the remaining eight video sequences (FIR1-test dataset) are used to evaluate the performance of a pedestrian detection system in the FIR1 dataset. It should be noted that the optimized parameters in FIR1-parameter-optimazation dataset will be used to test the video sequences in FIR1-test dataset; (2) The FIR2 is captured in seven different road sessions, corresponding to seven video sequences in total and each of which contains different numbers of frames. It contains 9065 one-channel full-size images and 3902 pedestrians in total. Amongst the seven video sequences which are named as FIR2-seq01 to FIR2-seq04 in Table 1, 3 video sequences (FIR2-parameter-optimazation dataset) are randomly selected for optimizing parameters, and the remaining four video sequences (FIR2-test) are used to evaluate the performance of a pedestrian detection system in FIR2 dataset. It should be pointed that the optimized parameters in FIR2-parameter-optimazation dataset will be used to test the video sequences in FIR2-test dataset. The details of FIR1 and FIR2 video sequence are shown in Table 1. As for the FIR2 dataset, because it is a public dataset and it does not contain the season information, so we cannot give out its season information. In the Detection Dataset, all the pedestrians whose heights are higher than 20 pixels and who are fully visible or less than 10% occluded have been labeled manually. Examples from this dataset are shown in Figure 15.

**Table 1:** Details of FIR1 and FIR2 video sequences.

Sequence Name	Total Frames	Annotated Pedestrians	Captured Date	Season	Remark
FIR1-seq01	44	25	26 November 2011	Winter	Optimizing parameters
FIR1-seq02	27	22	26 November 2011	Winter	Test video
FIR1-seq03	235	16	26 November 2011	Winter	Test video
FIR1-seq04	269	83	28 May 2012	Summer	Test video
FIR1-seq05	363	31	28 May 2012	Summer	Test video
FIR1-seq06	352	72	28 May 2012	Summer	Optimizing parameters
FIR1-seq07	527	263	16 August 2012	Winter	Test video
FIR1-seq08	489	477	16 August 2012	Winter	Test video
FIR1-seq09	305	148	16 August 2012.	Winter	Optimizing parameters
FIR1-seq10	482	88	August 2012	Winter	Test video
FIR1-seq11	847	286	10 January 2013	Winter	Test video
FIR2-seq01	945	154	-	-	Optimizing parameters
FIR2-seq02	540	244	-	-	Test video
FIR2-seq03	156	125	-	-	Optimizing parameters
FIR2-seq04	1459	758	-	-	Optimizing parameters
FIR2-seq05	1001	35	-	-	Test video
FIR2-seq06	3075	2586	-	-	Test video
FIR2-seq07	1889	0	-	-	Test video

### 6.2. Performance Evaluation Criterion

As what has been used in [3,38], we evaluate the system-level accuracy performance based on comparing all the positive detection windows (*DW_dt_*) in a given frame with the set of ground-truth windows (*DW_gt_*) in the same frame using the “Pascal Condition” [39] in Equation (15), when *φ* > 0.5, a *DW_dt_* matches with a *DW_gt_* that has not been matched before, it is counted as a correctly detected pedestrian, but if a *DW_dt_* doesn’t match any of the *DW_gt_*s, it is counted as a false alarm. Then on this basis, two metrics, *i.e.*, Detection Rate (*DR*) and False Alarm Rate (*FAR*), which are defined in Equations (16) and (17) respectively, are used to measure the system-level accuracy performance of a pedestrian detection system on the Detection Dataset:

(15) $$\varphi =area(DW_{dt}\cap DW_{gt})/area(DW_{dt}\cup DW_{gt})$$

(16) $$DR=N_{D}/N_{A}$$

(17) $$FAR=N_{FP}/N_{F}$$

where *N_D_* is the number of correctly detected pedestrians, *N_A_* is the number of annotated pedestrians, *N_FP_* is the number of false alarms and *N_F_* is the number of tested frames.

In order to get more intuitive comparison, the system-level accuracy is also evaluated by means of a Receiver Operating Characteristic (ROC) curve which quantifies the trade-off between *DR* and *FAR* by varying the decision threshold. In addition, ROC is also utilized for the accuracy performance evaluation of a classifier. The ROC curve is intuitive, but it does not quantify the overall system level accuracy with a value, so we also use the Log-Average Miss Rate (*LAMR*) as used in reference [15] to summarize detector performance. The *LAMR* is computed by averaging miss rate at five *FAR* rates evenly spaced in log-space in the range of 0.1 to 0.5, and a lower *LAMR* means a higher overall system level accuracy. Besides, in order to evaluate the processing speed of the detection system, we compute the Average Processing Frames per Second (*APFS*).

### 6.3. Evaluation of Head Detection Filter

**Figure 16:**
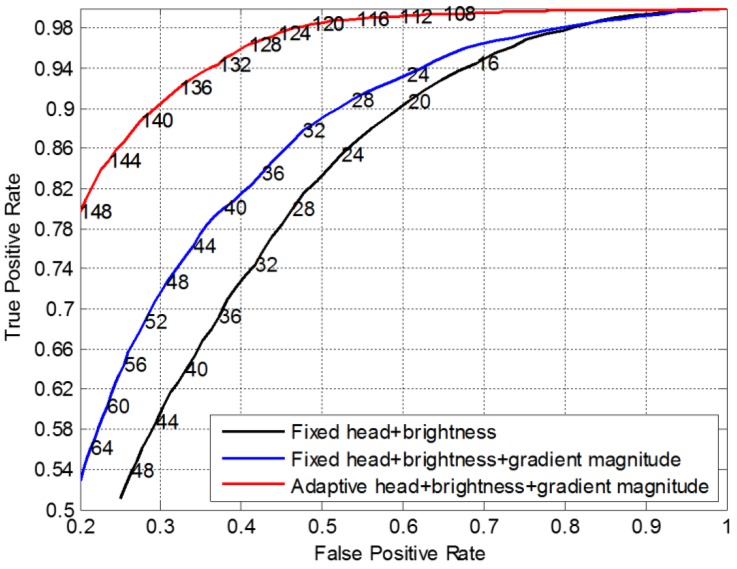
Accuracy performance comparison of different head detection filters.

In this section, not only is the performance of the proposed head detection filter evaluated, but also the contribution of the adaptive head location step and the additional gradient magnitude information are evaluated, respectively. Besides, the classification threshold of the head detection filter is optimized at the same time. More specifically, we perform our head detection filter on the Classification Dataset, and use the ROC curve to evaluate the classification performance, as the red curve shown in Figure 8. Based on this curve, on the one hand, in order to optimize the classification threshold of the head detection filter, the corresponding classification thresholds are drawn in the legend. As one can see, when the classification threshold is 120, the *DR* is 98% and the *FAR* is 50%, so this classification threshold has a rather high *DR* and can filter half of the false alarms, therefore, we chose 120 as the optimal classification threshold. On the other hand, in order to evaluate the contribution of the adaptive head location step and the additional gradient magnitude feature image respectively, the results of when the adaptive head location or the additional gradient magnitude feature image is not applied are also presented in Figure 16, which corresponds to the blue curve and the black one in the legend respectively, and the black curve corresponds to the head detection method of [34]. These experiments demonstrate the contribution of adaptive head location step and the additional gradient magnitude feature image proposed in this paper, and show that our head detection filter owns the best classification accuracy.

### 6.4. Performance Evaluation of the State-of-the-Art Features

**Figure 17:**
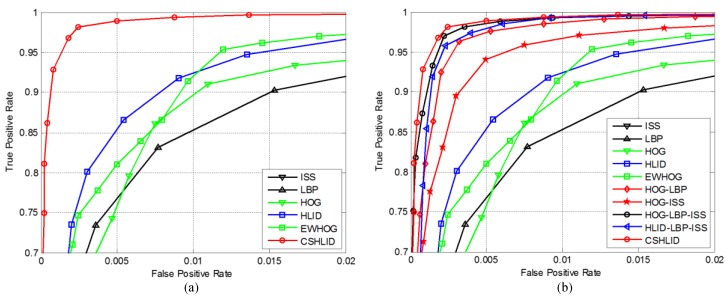
Accuracy performance comparison of classifiers using different features on FIR samples: (**a**) on single features; and (**b**) on hybrid features. Higher curves indicate better performance.

In order to evaluate the state-of-the-art features *versus* our proposed CSHLID feature, different features and a hybrid of features combined with the SVM learning algorithm are tested. On the one hand, we verify and compare the representation ability of six kinds of single features. The features include ISS [12], LBP [25], HOG [23], HLID [24], EWHOG [3] and the proposed CSHLID feature. Figure 17a shows the ROC curves of various features when a ten times ten-fold cross-validation is performed on a test set of 4800 pedestrian and 5000 non-pedestrian FIR samples. The experiment results demonstrate that the proposed CSHLID feature outperforms the state-of-the-art features, *i.e.*, at least 10% true positive improvement at 0.005 false positive per window. On the other hand, we also compare our CSHLID feature with the concatenation of some of the single features in the same experiment setting, and the experiment result is shown in Figure 17b, where “HOG-LBP” in the legend denotes the concatenation of HOG feature and LBP feature, and “HOG-ISS”, “HOG-LBP-ISS” and “HLID-LBP-ISS” denote the corresponding features concatenate in the same way. Figure 17b shows that our CSHLID feature also keeps the highest accuracy even compared with the concatenation of some state-of-the-art features. Accordingly, experiment result in Figure 17 demonstrates that our CSHLID feature is more suitable for FIR pedestrian classification than lots of well-known pedestrian detection features in terms of accuracy.

### 6.5. Parameter Optimization

**Figure 18:**
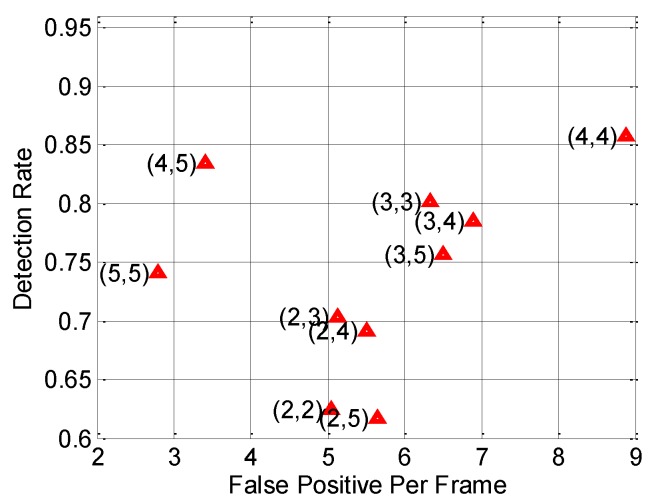
Performance of using diverse setting of the size (*M* × *N* pixels) of the mask for the morphological operations on FIR1-parameter-optimazation dataset.

In this section, the penalty factor *C* for the SVM learning algorithm, the size (*M* × *N* pixels) of the mask for the morphological operations and the matching threshold *T* for MFAM are optimized separately. The penalty factor *C* is optimized by 10-fold validation on the corresponding training set using the well-known Open Source Computer Vision Library (OpenCV) tool [40] (version 2.3.1), and the optimal value of *C* returned by this tool is 0.5062. Because the resolution of an image on FIR1 dataset is almost five times higher than that on FIR2’s, the size (*M* × *N* pixels) of the mask and the matching threshold *T* should be optimized separately on the FIR1-parameter-optimazation dataset and FIR2-parameter-optimazation dataset, where the FIR1-parameter-optimazation dataset contains FIR1-seq01, FIR1-seq06 and FIR1-seq09 listed in Table 1, and the FIR2-parameter-optimazation dataset contains FIR2-seq01, FIR2-seq03 and FIR2-seq04 listed in Table 1. In order to optimize the size of the mask, based on the segmentation results obtained from the interlaced segmentation mentioned in Section 4.1, we compute the *DR* and the *FPPF* on FIR1-parameter-optimazation dataset using diverse setting of the mask size, with the result shown in Figure 18. 

**Figure 19:**
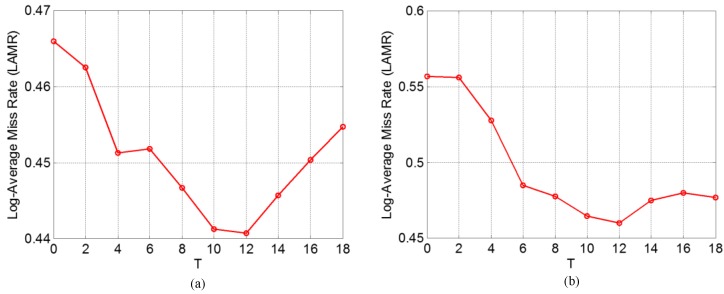
Performance of using diverse setting of *T*: (**a**) on FIR1-parameter-optimazation dataset; and (**b**) on FIR2-parameter-optimazation dataset.

In consideration of the problem that if a pedestrian candidate does not generate successfully, then this pedestrian cannot be detected by the follow-up modules, we choose the mask size which is of the highest *DR*. Therefore, the optimal mask size is (4, 4) because it has the highest *DR* as shown in Figure 18. For the FIR2-parameter-optimazation dataset, we adopt the same method to find the optimal mask size as adopted in the FIR1-parameter-optimazation dataset, but we find that the optimal choice is not to use the morphological operations in this dataset, because the resolution of an image in the FIR2 dataset is almost five times smaller than that in FIR1, which leads to the fact that our morphological operations are not suited for the small-scale pedestrians appearing in the FIR2 dataset, so we do not apply the morphological operations when testing our proposed method on the FIR2 dataset. In order to optimize the matching threshold *T* for MFAM, Figure 19 presents the trend of performance change with respect to diverse settings of *T* on the corresponding parameter optimization datasets, which demonstrates that the optimal *T* should be 12. Because of the existence of camera/pedestrian motion, a *T* of too low value may fail to match the pedestrians, while a *T* of too high value may lead to an incorrect match with a non-pedestrian. As a result, the optimal *T* is 12 for both the FIR1 and FIR2 datasets.

### 6.6. Performance Evaluation of the Proposed Pedestrian Detection System

**Figure 20:**
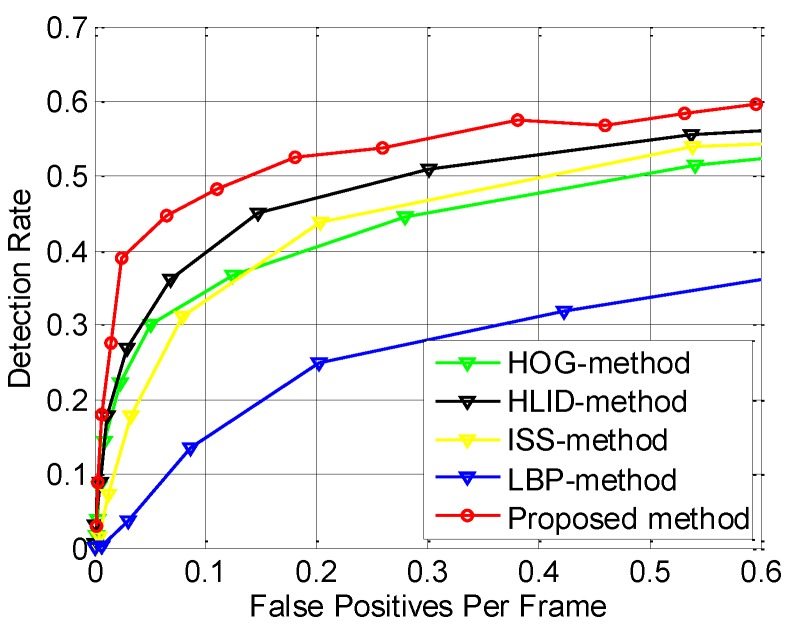
Accuracy performance comparison of various pedestrian detectors on FIR1-test dataset.

In order to evaluate the system-level performance of the proposed pedestrian detection system, we test our proposed system (denoted by “Proposed method”) on the FIR1-test dataset. The ROC result is the red curve in Figure 20. Furthermore, in order to evaluate the contribution of the two novel candidate filters, the novel CSHLID feature, and MFAM algorithm, we replace the CSHLID feature with some state-of-the-art features, respectively, and remove the two candidate filters and MFAM module in the whole system, then we obtain four pedestrian detection systems (HOG-method, HLID-method, ISS-method, and LBP-method) for comparison. The comparison results are shown in Figure 20, where as expected, our proposed method has the highest accuracy performance.

**Figure 21:**
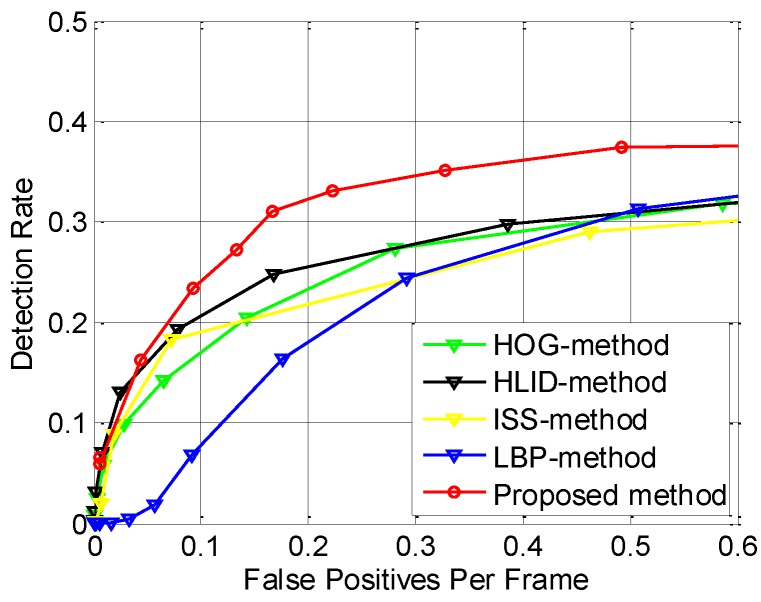
Accuracy performance comparison of various pedestrian detectors on FIR2-test dataset.

In addition, to make sure that the improvement is not dataset-specific, we perform verification on the FIR2-test dataset. The results are shown in Figure 21, where the improvement is similar to that in Figure 20, thus proving that the improvement of the performance is not dataset-specific.

There are three reasons for the improvement: (a) the two candidate filters proposed in this paper reduce the *FAR* by rejecting the non-pedestrian candidates; (b) the MFAM increases the *DR* by utilizing the spatial-temporal continuity of the pedestrians; (c) our proposed CSHLID feature has stronger representation ability than the state-of-the-art features, which not only helps to reduce the *FAR* but also helps to increase the *DR* of the system. As a result, the *FAR* has been reduced and the *DR* has been increased, so the ROC performance has been improved.

The ROC curves in Figure 20 and Figure 21 are intuitive, but they do not quantify the overall system level accuracy with a value, so we also compute the *LAMR* to summarize the performance of various detection methods. In addition, we also give out the *DR* when the *FAR* is 0.2, as shown in Table 2, where “ISS-method”, “LBP-method”, “HOG-method” and “HLID-method” in the table denote the detectors featured in ISS, LBP and HOG combined with lin-SVM, respectively. Besides, the “Proposed method” denotes the pedestrian detection method proposed in this paper. 

**Table 2:** Accuracy performance evaluation on various detection datasets.

Method	FIR1-Test		FIR2-Test		FIR1-Test-Summer		FIR1-Test-Winter	
	*LAMR*	*DR*	*LAMR*	*DR*	*LAMR*	*DR*	*LAMR*	*DR*
ISS-method [12]	0.5343	0.4360	0.7433	0.2396	0.5391	0.4493	0.5298	0.4380
LBP-method [25]	0.7298	0.2477	0.7743	0.1834	0.9077	0.0636	0.6978	0.2802
HOG-method [23]	0.5562	0.4111	0.7139	0.2392	0.6152	0.3482	0.5477	0.4228
HLID-method [24]	0.5019	0.4778	0.7260	0.2593	0.6057	0.3691	0.4790	0.5038
Proposed method	0.4567	0.5301	0.6678	0.3254	0.6482	0.3264	0.4316	0.5540

**Figure 22:**
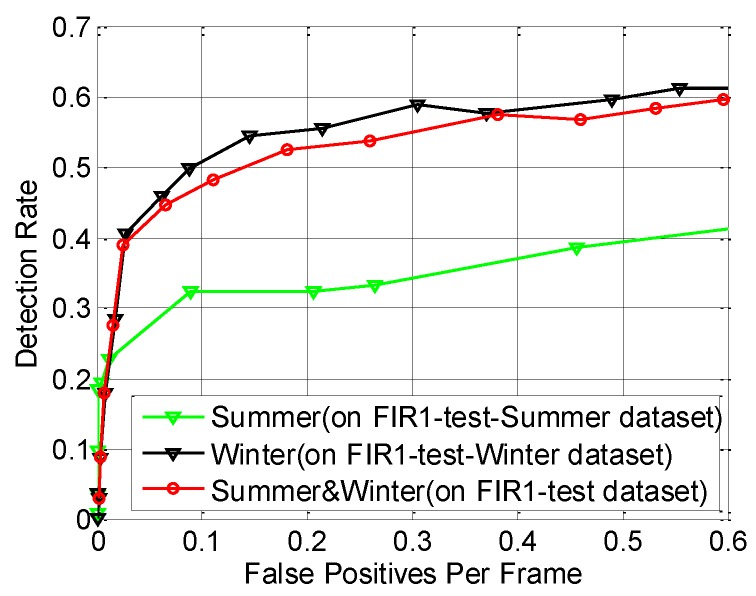
Performance evaluation of our proposed method on datasets captured from different seasons.

As shown in Table 2, after using our novel CSHLID feature, two novel candidate filters and the MFAM approach, our method gets the lowest (best) *LAMR* value on both FIR1-test (which denotes all the test sequences in the FIR1 dataset) and FIR2-test (which denotes all the test sequences in the FIR2 dataset) datasets. The average improvement is 12% on the FIR1-test dataset and 7% on the FIR2-test dataset. The lowest *LAMR* is reduced from 50% to 45% on the FIR1-test dataset, and 71% to 66% on the FIR2-test dataset. In summary, the average improvement is 9% on the FIR1-test and FIR2-test datasets. At the same time, we also list the *DR* when the *FAR* is 0.2, and our method also has the optimal performance compared with the other four methods. In addition, because the detection performance in FIR sequences may probably be different for a summer dataset and a winter dataset because of the thermal radiation of pedestrians, we also evaluate the performance difference when different methods are applied. The experimental results are also shown on Table 2, where the “FIR1-test-Summer” means all the sequences of FIR1-test dataset captured in summer as shown in Table 1, and the “FIR1-test-Winter” means all the sequences of FIR1-test dataset captured in winter as shown in Table 1. In order to get a more intuitive comparison of our proposed method on datasets captured from different seasons, the ROC performance of our method on datasets captured in different seasons is shown in Figure 22. As expected, since the pedestrians are warmer than the background in winter and less background heat sources will interfere the detection of pedestrians, the system performance on dataset captured in winter is higher than that in summer for the five methods compared in Table 2.

In order to evaluate candidate filters and the MFAM, we remove the candidate filters and the MFAM from our proposed method, respectively, and test the result on the FIR1-test and FIR2-test dataset, respectively. More specifically, the experiments show that, on the FIR1-test dataset, the LAMR will increase from 45.67% to 45.86% when the candidate filters are removed, and the LAMR will increase from 45.67% to 49.43% when the MFAM is removed; on the FIR2-test dataset, the LAMR will increase from 66.78% to 68.43% when the candidate filters are removed, and the LAMR will increase from 66.78% to 69.49% when the MFAM is removed. In summary, both the candidate filters and the MFAM produce an improvement on the overall system performance.

**Figure 23:**
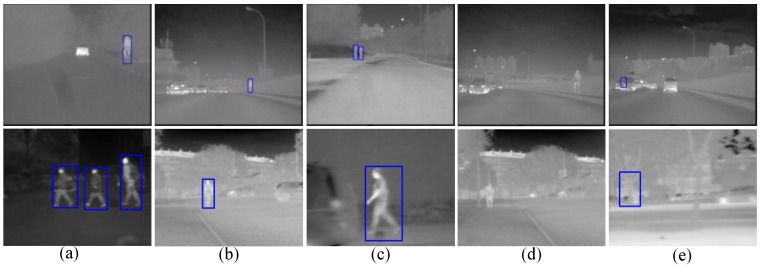
Examples of detection results. The first row shows the detection examples from FIR1-test dataset, the second row shows the detection examples from FIR2-test dataset; (**a**)–(**c**) are examples of successful detection in various scenarios and uneven road environments; (**d**) is examples of missed detections; and (**e**) is examples of false alarms.

Some examples of detection results from our proposed system are shown in Figure 23. As one can see, the proposed method adapts to not only the variation of backgrounds but also the variation of pedestrians. Examples of missed detections are shown in Figure 23d, where most of the undetected pedestrians suffer from their non-uniform brightness or the interference of bright background. Besides, examples of some false alarms are shown in Figure 23e, where most of the false alarms are caused by their similar shape to pedestrians. To name a few, streetlights, traffic signs, part of a vehicle, buildings and tree trunks.

Moreover, the proposed pedestrian detection system has some additional advantages: (a) the system allows the camera to work in uneven road environments and to have a different installation height for a vehicle-mounted application; (b) because our proposed system is implemented using the C programming language and is of high efficiency, the system is suitable to be transplanted to an embedded system to implement a practical pedestrian detection system for ADAS, besides, this advantage also provides room for further improvement; (c) because the system is based on a monocular camera, it has the potential to integrate other cameras to further increase the performance.

### 6.7. Processing Time Evaluation

To evaluate the processing speed of the related detection systems, we calculate the *APFS* of the six pedestrian detection systems of Table 2. All the systems are tested on a Windows 7 platform (×64) with a 2.53 GHz, i5 dual-core processor, 3 GB DDR computer using C programming language. As for our proposed pedestrian detection system, the *APFS* on the FIR1-test and FIR2-test dataset is about 31 and 63, respectively. This difference mainly results from the fact that the resolution of an image in the FIR1-test dataset is almost five times higher than those in the FIR2-test’s. The experimental result demonstrates that although the processing speed has decreased slightly on the above two datasets, our method also works in real time while the *LAMR* has increased at least 4% and 9% on average on the FIR1-test and FIR2-test dataset. The result demonstrates that the accuracy improvement is significant and the impact on real-time performance is relatively minor. This has practical importance because fast detection rates and low computational requirements are of the essence for ADAS [27].

## 7. Conclusions

This paper has introduced a robust and real-time pedestrian detection system for ADAS based on a monocular FIR camera. This system has four modules in a cascade coarse-to-fine fashion, and each module utilizes features from the FIR pedestrians and backgrounds to successively distinguish the pedestrians from the cluttered background.

In order to improve the robustness and real-time performance of current methods which are only based on high-dimensional features, some novel approaches have been proposed in this paper, including: (1) two efficient candidate filters are proposed to reduce the false alarms; (2) a novel pedestrian detection feature is designed to deal with the enormous variance in pedestrians’ size and appearance; (3) a MFAM approach is introduced in order to increase the detection rate. 

Large-scale experiments under various scenarios indicate that the system is robust and efficient, and suitable for real-time practical applications. The satisfactory performance is due to the combination of the effective candidate filters and the improved appearance-based detector, and proper utilization of the spatiotemporal continuity of pedestrians, which can benefit from the strengths of different techniques and overcome their respective disadvantages. It is usually believed that when one improves the pedestrian detection accuracy, the price to pay is to sharply increase computational costs, but we have shown that this is not necessarily so.

Regarding future work, firstly, more research is needed to reduce the partly segmented pedestrians, so that the missed detections can be reduced. Secondly, we plan to explore the scene knowledge contained in typical traffic scenes, and integrate them to the system in this paper to further enhance the robustness. Thirdly, as our system has excellent real-time and accuracy performance, we are going to implant our system into an embedded system to form a practical pedestrian protection product.
